# Assessing the plasmonics of gold nano-triangles with higher order laser modes

**DOI:** 10.3762/bjnano.3.77

**Published:** 2012-10-04

**Authors:** Laura E Hennemann, Andreas Kolloch, Andreas Kern, Josip Mihaljevic, Johannes Boneberg, Paul Leiderer, Alfred J Meixner, Dai Zhang

**Affiliations:** 1 Institute of Physical and Theoretical Chemistry, University of Tübingen, Auf der Morgenstelle 15, 72076 Tübingen, Germany; 2 Department of Physics, University of Konstanz, Universitätsstrasse 10, 78457 Konstanz, Germany

**Keywords:** Fischer pattern, higher order laser modes, localised surface plasmons, near field, surface-enhanced Raman scattering

## Abstract

Regular arrays of metallic nano-triangles – so called Fischer patterns – are fabricated by nano-sphere lithography. We studied such gold nano-triangle arrays on silicon or glass substrates. A series of different samples was investigated with a parabolic mirror based confocal microscope where the sample is scanned through the laser focus. By employing higher order laser modes (azimuthally and radially polarised laser beams), we can excite the Fischer patterns using either a pure in-plane (x,y) electric field or a strongly z-directional (optical axis of the optical microscope) electric field. We collected and evaluated the emitted luminescence and thereby investigated the respectively excited plasmonic modes. These varied considerably: firstly with the light polarisation in the focus, secondly with the aspect ratio of the triangles and thirdly with the employed substrate. Moreover, we obtained strongly enhanced Raman spectra of an adenine (sub-)monolayer on gold Fischer patterns on glass. We thus showed that gold Fischer patterns are promising surface-enhanced Raman scattering (SERS) substrates.

## Introduction

The interaction of light and matter is especially intriguing in those cases where the size of the matter particle is comparable to or smaller than the wavelength of the light. When illuminating metallic nano-particles with light of a matching frequency, particle plasmons (also called plasmon polaritons or localised surface plasmons, LSP) can be created and investigated [1]. These quantised collective oscillations of the electrons in the metal have been at the centre of a relatively recent field of study called plasmonics.

In addition, such particle plasmons yield an evanescent electromagnetic field at the outline of the nano-particles. This so-called near field can be highly enhanced and strongly confined in space. So far, the near field at the outline of nano-particles in general [2] and nano-triangles in particular [3–6] has been investigated both directly and indirectly.

Likewise, the plasmonic behaviour of metallic nano-triangle *arrays*, namely Fischer patterns [7] made by colloid lithography, has been studied to some extent. Optical extinction spectroscopy [8], confocal microscopy [9], photopolymerisation [6] and near-field ablation [3,10–11] have been performed. It was found that the material of the triangles plays a distinct role [9], as do the triangles' edge length and height [3,8,10–11] and the material of the underlying substrate [12].

Additionally, nano-particles and nano-particle arrays are long known to yield particle-enhanced Raman spectra [13–15]. Hence, there have also been several works [16–18] using the near field of Fischer patterns for surface-enhanced Raman scattering (SERS [19–20]). However, to our knowledge, no investigations of Fischer patterns by higher order laser modes have been reported so far.

Here we present the investigation of gold nano-triangle Fischer patterns with a custom built confocal microscope. The use of higher order laser modes allows for the tuning of the polarisation in the laser focus so that it can be either perpendicular to the sample surface or parallel to it. The parameters varied in the Fischer patterns were the size of the triangles, their height and the substrate material (which was either silicon or glass). Using integrated shear-force microscope, we can collect the topography from the sample surface and compare the position of the triangles with the confocally observed luminescence patterns. Ultimately, we also achieved surface-enhanced Raman spectroscopic studies of adenine molecules adsorbed on such gold nano-triangles.

## Experimental

**Figure 1:**
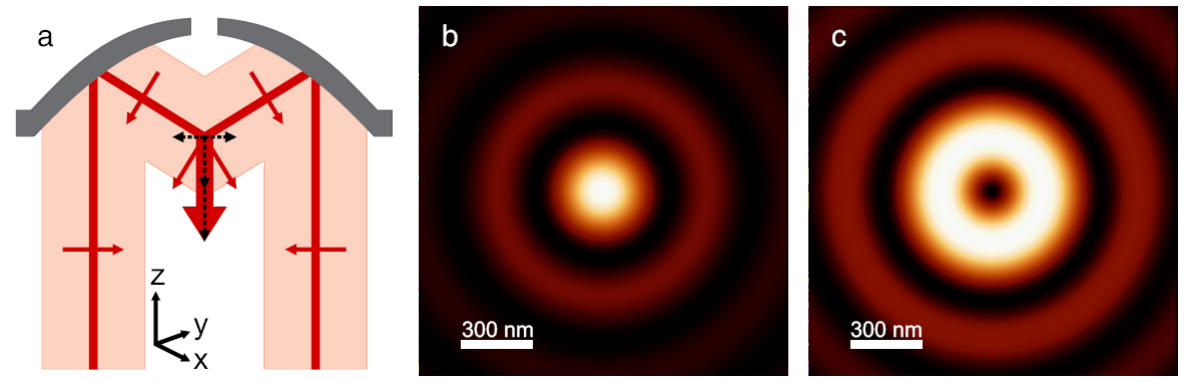
Focusing higher order laser modes with a parabolic mirror. (a) A sketch illustrating how a radial mode yields a strong field distribution in z-direction (optical axis of the microscope) in the focus of a parabolic mirror. The small red arrows depict the direction of the electric field; the dashed black arrows show their vector components. In the focus, the E-field components in x and y cancel each other out while the components in z sum up: large red arrow. (b) and (c): Calculation of the free-space field distribution (x,y plane-section) of higher order laser modes focused by a parabolic mirror; laser wavelength λ = 632.8 nm. (b) Focus of the radial donut mode. Only |*E*_z_|^2^, i.e., the electric field intensity normal to the sample surface is depicted; the components parallel to the sample surface are considerably weaker. (c) Focus of the azimuthal donut mode. The total intensity is depicted and corresponds to that of the field in x and y since *E*_z_ is zero. The field polarisation is azimuthal within the bright focal ring.

Our custom-built parabolic mirror based near-field optical microscope has been described previously [21–22]. The Gaussian beam of a helium-neon laser (λ = 632.8 nm) is transformed by a mode converter to generate higher order laser modes: either a radially or an azimuthally polarised donut mode [23–24]. This mode is then focused onto the sample surface by a parabolic mirror of high numerical aperture (NA = 0.9998). The radial mode results in a strong longitudinal electric field in the focus (normal to the sample surface, i.e., a z-polarisation), while the azimuthal mode yields an electric field purely in the x,y-plane of the focus (Figure 1). In radial mode, the focal spot has a full width at half maximum (FWHM) of approximately 260 nm [24]. In azimuthal mode, the intensity in the focus is ring-shaped with an outer FWHM of 640 nm and an FWHM of the ring itself of 220 nm [25]. The laser power in the focus is 250 μW. The same parabolic mirror also serves as the collecting element for the scattered luminescence coming from the sample. This scattered light is separated from the incoming laser beam by a beam splitter. Subsequently, two consecutive notch filters block the laser wavelength in order to select only the inelastically Stokes scattered light. This signal is finally directed either onto an avalanche photodiode (APD) or onto a grating spectrometer coupled with a liquid nitrogen cooled CCD camera.

The samples were prepared by colloid lithography. Via drying of a strongly diluted colloidal suspension, a self-assembled monolayer of polystyrene spheres was created on a substrate piece of either silicon (commercial silicon wafer) or glass (commercial glass slide by Menzel-Gläser). A thin gold film was then evaporated with the polystyrene colloids acting as a mask on the substrate. Finally, the colloids were removed with a piece of adhesive tape. The resulting sample is a regular hexagonal array of gold triangles on the substrate, which, as already mentioned above, is often referred to as a Fischer pattern [7].

**Figure 2:**
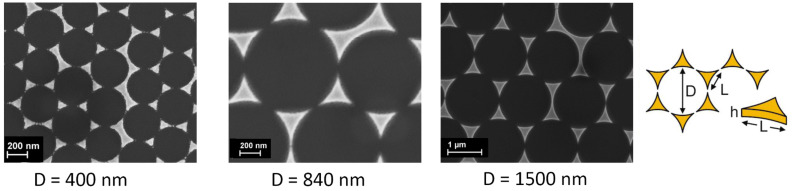
SEM images of gold triangle arrays on silicon substrates which are fabricated using polystyrene spheres of different diameters. The sketch on the right shows the used denominations to characterise the triangle array.

Polystyrene spheres of diameters *D* between 200 and 1500 nm were used. The film height *h* of the evaporated gold was varied between 40 and 200 nm (see Figure 2b for the denominations). Not all combinations of sphere size and film thickness could be produced since the film thickness has to be distinctly smaller than the polystyrene spheres in order to obtain flat triangles. Thicker triangles would have the form of a truncated pyramid [26]. By shear-force topographical measurements [27], we found the triangles to have an edge length *L* of approximately 80% of a sixth of the circumference of the spheres used; this value confirms the findings by Gonçalves et al. [9]. Figure 2 shows SEM images of gold triangle arrays on silicon substrates which are fabricated employing polystyrene spheres of different diameters.

For the SERS investigations, we prepared a solution of 10^−6^ M adenine (Sigma-Aldrich) in triple distilled water. A 30 µL droplet was then put on the sample and after one hour of incubation gently soaked off with a lint-free tissue without touching the surface. Plenty of water was used to rinse the surface in order to get rid of the physically adsorbed multilayers.

## Results and Discussion

### 1. Topography

Investigating the triangle arrays with the shear-force topographical method implemented in our setup [22] and SEM characterisations, we find well aligned triangles interrupted by slightly larger gold structures. The latter result from dislocations within the self-assembled monolayer of the polystyrene spheres [9–10].

### 2. Confocal luminescence patterns of gold Fischer patterns

We performed confocal scanning images of six Fischer patterns of different triangle aspect ratio on silicon and of three on glass, all of them both in radial and in azimuthal mode. With these higher order laser modes, we can pointedly excite specific particle plasmon modes in the nano-triangles. Strong plasmonic excitation leads to strong luminescence. The recorded luminescence images therefore reflect the excitations of local particle plasmons.

#### 2a. Fischer Patterns on silicon excited by radial polarisation

**Figure 3:**
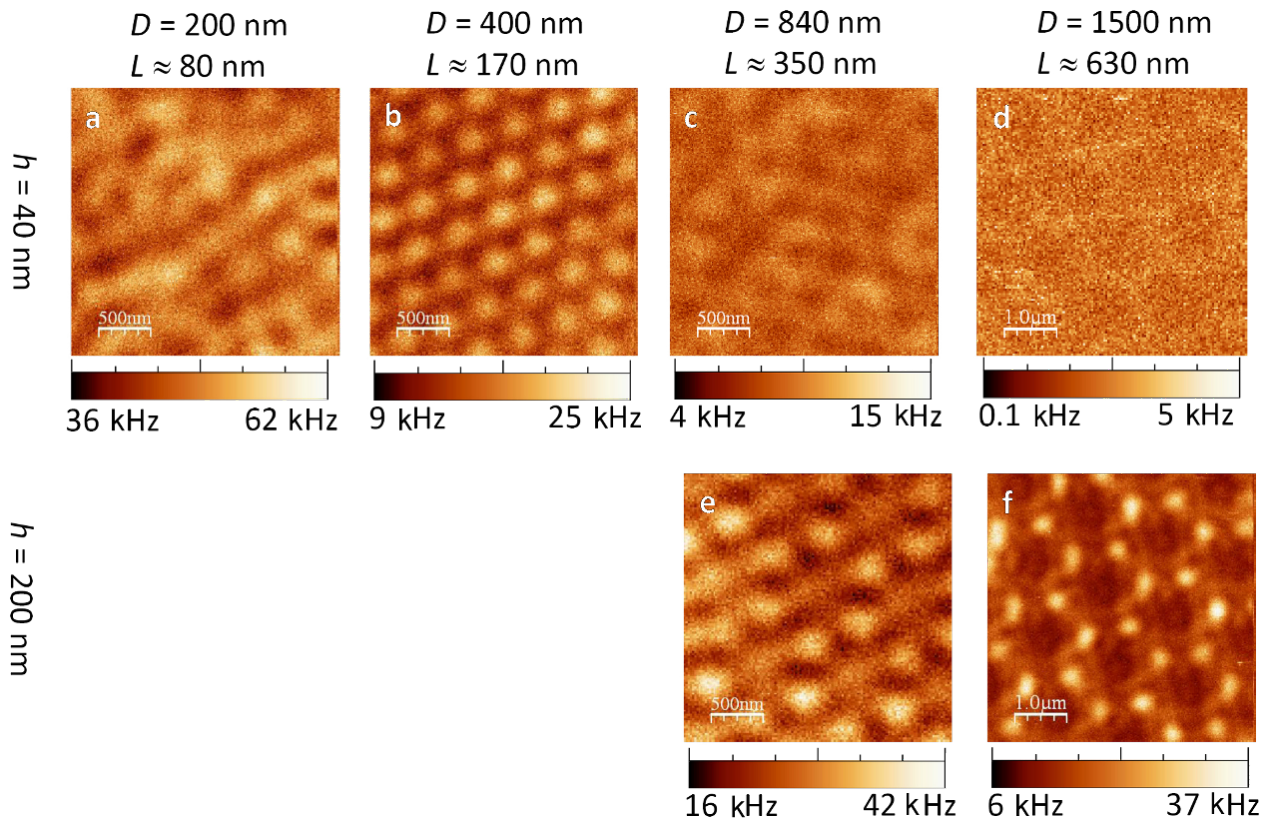
Confocal luminescence images of gold Fischer patterns on a silicon substrate. The images were recorded by scanning the sample through the focus of a *radially polarised laser beam*. Note the twice as large dimensions of images (d) and (f).

Most investigated thin (*h* = 40 nm, Figure 3a–d) nano-triangles on silicon yielded an extremely weak luminescence contrast. In radial mode, the triangles with *L* ≈ 170 nm (Figure 3b) were the only ones to show a clear pattern. Due to their hexagonal layout and inter-distance, we believe that each of the bright spots marked the centre of a former bead position.

The thicker triangles with *h* = 200 nm yielded clearer contrasts. The Fischer pattern with *L* ≈ 350 nm showed bright spots of triangular shape (Figure 3e). These had to be located at the same places as the gold nano-triangles. However, their orientation was turned by 60° with respect to that of the gold triangles.

Eventually, the largest thick triangles (*h* = 200 nm, *L* ≈ 630 nm, Figure 3f) showed a regular pattern of bright dots with some additional structures in-between. Each bright spot can be assigned quite straightforwardly to the location of a gold triangle. The first maximum ring of the radial focal intensity (recall Figure 1b) is very likely to be the cause of the additional luminescence structures.

#### 2b. Fischer patterns on silicon excited by azimuthal polarisation

**Figure 4:**
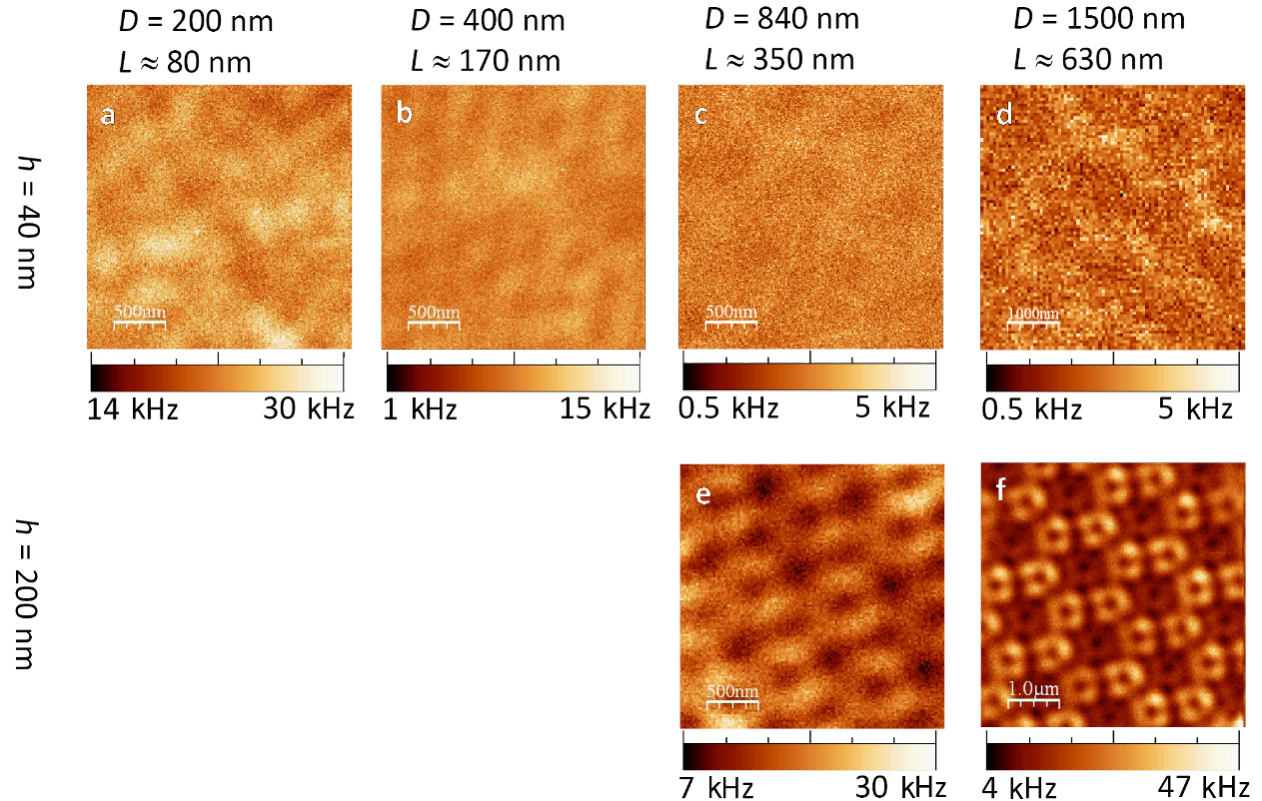
Confocal luminescence patterns of gold nano-triangles on silicon excited by an *azimuthally polarised laser beam*. Note the larger scales of (d) and (f).

In azimuthal donut mode illumination, the thin triangles with *h* = 40 nm (Figure 4a–d) showed the same weak contrast as in the radial case. The thicker triangles with *h* = 200 nm showed clearer images. With the Fischer patterns of *L* ≈ 350 nm (Figure 4e), the confocal image showed a periodic structure. Its reoccurring sub-pattern resembled bright rods aligned to each other like the edges of a triangle. By comparing this luminescence structure to the most probable former position of the polystyrene beads, we found that each of these luminescing triangles was rotated by 60° against an actual gold triangle. The largest triangles with *h* = 200 nm, *L* ≈ 630 nm showed a regular pattern of bright hollow triangles (Figure 4f). Considering the hexagonal position of each nano-triangle, the brightest spots do not appear at the sharp tips nor at the triangle centres, but rather at the middle of their edges. This can be compared to what has been observed as the edge mode in nanoprisms [5].

#### 2c. Fischer patterns on glass excited by radial polarisation

**Figure 5:**
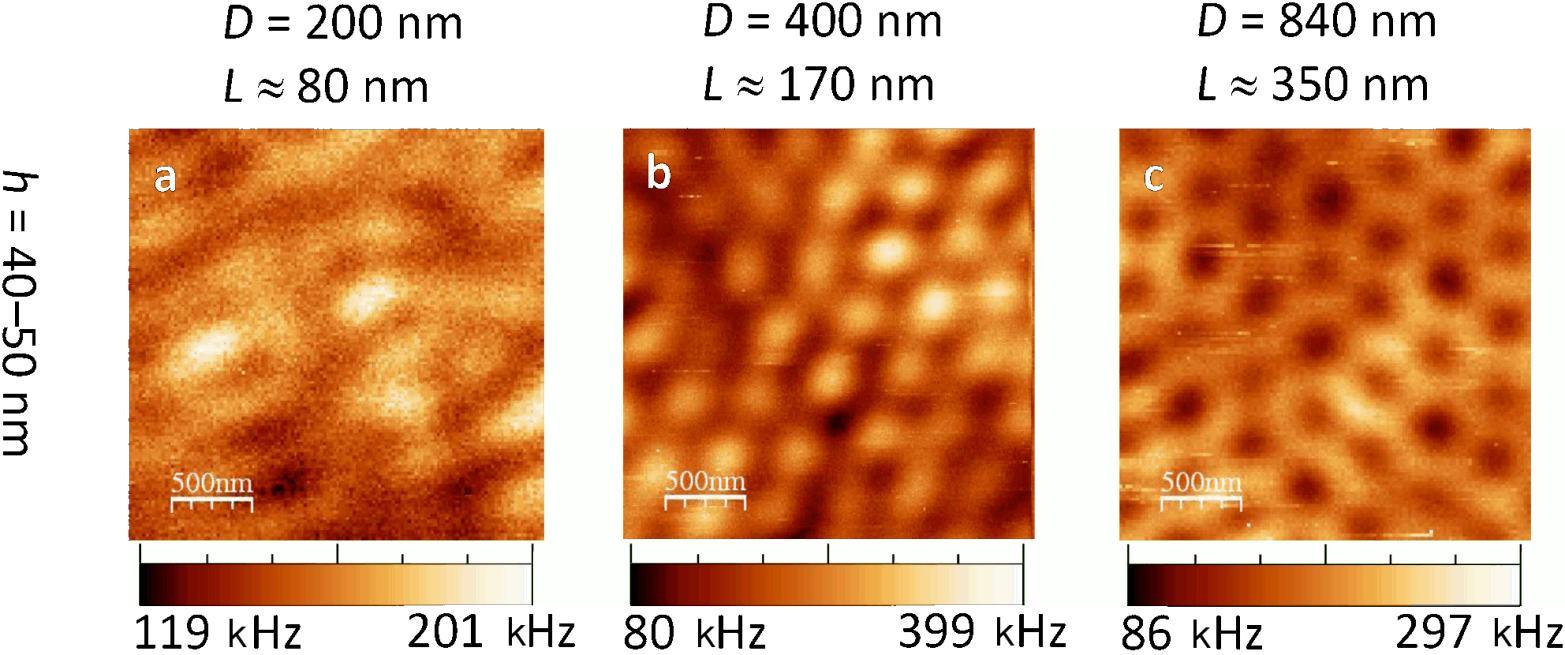
Confocal luminescence patterns of gold nano-triangles on glass excited by a *radially polarised laser beam*. In (a) and (b) the height of the triangles was *h* = 40 nm, whereas in (c) it was *h* = 50 nm.

**Figure 6:**
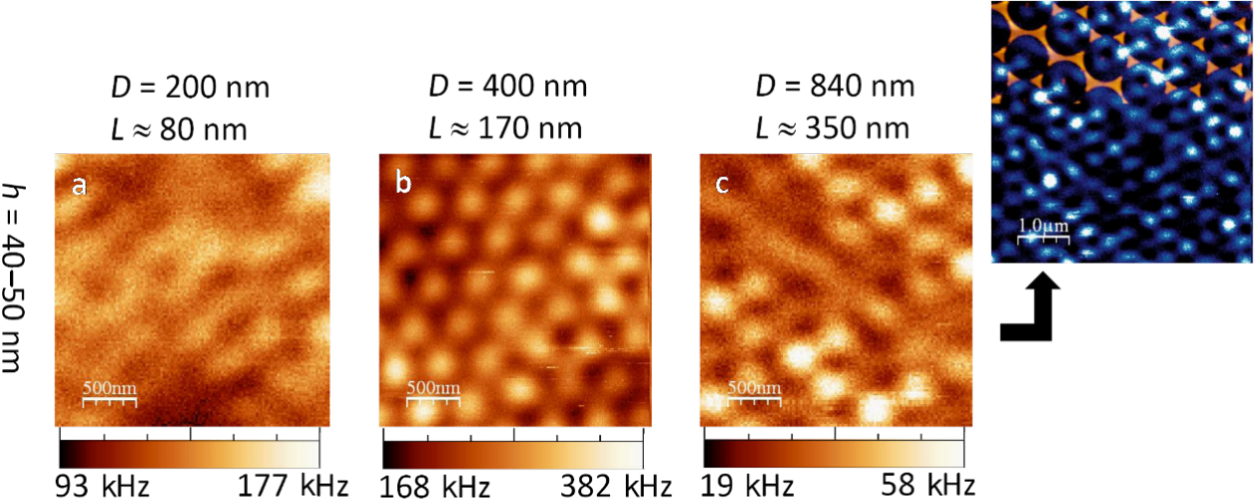
Confocal luminescence patterns of gold nano-triangles on glass excited by an *azimuthally polarised laser beam*. In (a) and (b) *h* = 40 nm, whereas in (c) *h* = 50 nm. The blue image was performed on the same sample as (c) and is an overlay of a topographical shear-force measurement (shown in blue) and a subsequent confocal optical measurement (shown in golden).

In both higher order laser modes, Fisher patterns on glass (Figure 5 and Figure 6) yielded a significantly stronger luminescence and a higher contrast than the triangles on silicon. On glass, it can be observed that the patterns varied considerably both with the edge length *L* and with the height *h* of the nano-triangles.

Regarding specifically the investigation in radial laser mode (Figure 5), it was not possible to recognise a regular pattern with the small triangles (*h* = 40 nm and *L* ≈ 80 nm, Figure 5a). This could be due to a poorly aligned monolayer of the small *D* = 200 nm polystyrene spheres used. Moreover, our focal size of 260 nm is much larger than these triangles and hence cannot resolve them.

The medium sized triangles (*h* = 40 nm, *L* ≈ 170 nm, Figure 5b) showed a hexagonal pattern of bright dots. The pattern resembled very much that of the gold triangles on silicon (Figure 3b). Like there, it seems that each bright dot marks the former centre of a polystyrene sphere.

The large, thin triangles (*h* = 50 nm, *L* ≈ 350 nm, Figure 5c) yielded a honeycomb structure of hexagonally connected lines. Taking a closer look, every second dark spot in a row was slightly larger than the others. These larger dark areas had an overlying hexagonal alignment with a mean distance of approximately 870 nm. Even though this is 4% more than the denoted polystyrene sphere size of *D* = 840 nm, we conclude that these larger dark spots mark the former sphere positions. Slight misalignments – as also observed topographically (Figure 2) – will always lead to a larger mean distance of their positions.

Provided that these larger dark areas are the former bead positions, we deduce from Figure 5c that each gold triangle yielded a bright threefold star where the beams were directed towards the edges of the triangle. The secondary dark areas were then located between two adjacent gold triangle tips. This can be explained by a strong plasmonic excitability at the centre of the nano-triangle by means of a plasmonic centre mode and additional excitability at the triangle edges by an edge mode [5]. These eigenmodes of plasmonic enhancement are dubbed according to the spots of strong field enhancement. This near field radiates into the far field which is the luminescence we then observe. However, we can make no definite comparison to other works with Fischer patterns since no other report of nano-triangle investigations by radially polarised laser beam is known to us.

#### 2d. Fischer patterns on glass excited by azimuthal polarisation

With the azimuthal laser mode (Figure 6), we partially observed different luminescence patterns of gold triangles on glass than in radial mode. The smallest triangles again showed no regularity in the intensity pattern (Figure 6a).

The medium sized triangles (Figure 6b) yielded a pattern of bright dots very similar to the investigation by radial laser mode. In fact, when scanning the same area consecutively in radial and azimuthal mode, it became evident that in both cases the exact same locations showed luminescence maxima. This is surprising, since we have to bear in mind the different focal patterns (recall Figure 1b,c). In contrast to radial mode, a bright spot in azimuthal mode is actually not located where it appears to be but somewhere in a radius of 530 nm next to it, considering the azimuthal focus has an intensity *minimum* in the centre. In addition, the average photoluminescence intensity of the medium sized triangles (Figure 6b) is the strongest among the three different sizes (Figure 6a–c).

Additionally, we placed our shear-force tip into the azimuthal focus and performed a topographical scan on this last sample directly after a confocal measurement. Since the azimuthal focus is isotropic in x and y, the luminescence pattern cannot affect the three gold triangle tips in different ways. However, in the overlay of topographical and confocal measurement (blue/golden image in Figure 6), this seems to be the case. We conclude that the tip had been shifted slightly with respect to the centre of the azimuthal focus. Nevertheless, we learn from this overlay that larger gold structures (e.g., joint nano-triangles, as resulting from misalignments in the polystyrene monolayer) yield areas of weaker confocal signal. We could confirm this by observations in many further measurements both in radial and in azimuthal mode. This proves that our confocal luminescence patterns depict particle plasmon enhancements. Continuous gold clusters cannot be excited as efficiently as the separated nano-triangles.

As a first conclusion, we found that the gold Fischer patterns on glass yielded luminescence patterns which were very different from those of the triangles on a silicon substrate. The contrast and luminescence intensity were considerably higher on glass. Only the patterns of the *h* = 40 nm, *L* ≈ 170 nm sized triangles in radial mode led to resembling patterns on silicon and glass (Figure 3b and Figure 5b). However, the photoluminescence intensity from Fischer patterns on glass is nearly 15 times stronger.

Turning to the Mie theory, we note that the dielectric constant of the medium surrounding the metal nano-particles plays a crucial role in describing its plasmonic resonances. In our case, the particles are flanked by two different media: air above and silicon or glass below them. While at 633 nm the refractive index of glass is roughly ε_glass_ ≈ 1.5, that of crystalline silicon is as high as ε_Si_ = 3.9 [28]. This great difference inevitably influences the plasmonic responses of Fischer patterns of the same geometry when located on these two different substrates.

Note that although silicon forms a natural glass-like oxide layer on its surface, this layer is extremely thin (in the order of 20 Å [29]). In comparison, a triangle's near field extends over some tens of nanometres. We can hence assume that the high refractive index of the pure silicon is mainly responsible for our observations.

#### 2e. Comparison with a theoretical convolution solution

**Figure 7:**
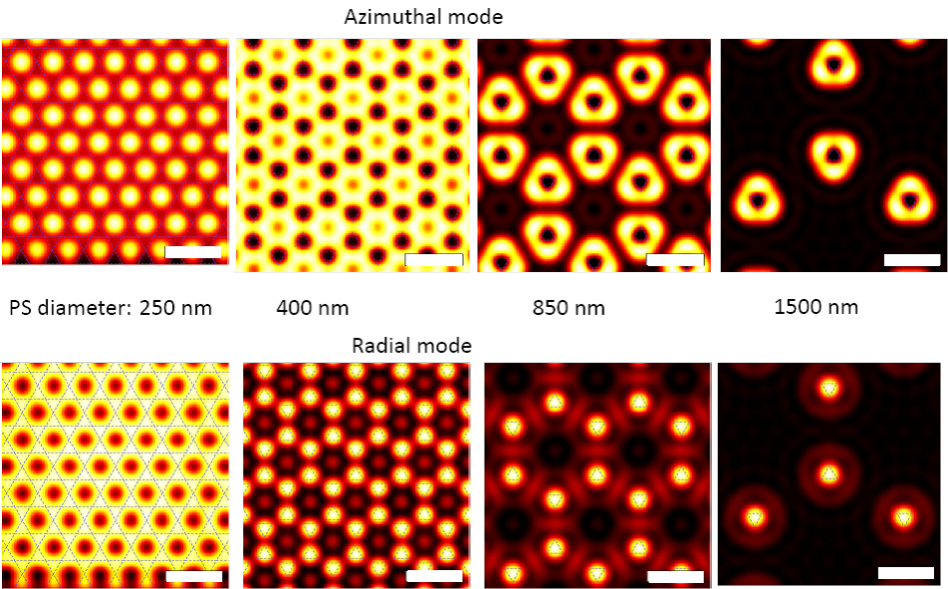
Calculated convolution of the intensity distribution in the azimuthal laser focus (upper row) and radial laser focus (lower row) with Fischer patterns of various sizes. The diameters of the PS spheres used for the Fischer pattern fabrication are indicated in between the two rows. The scale bars in all pictures indicate lengths of 1 micrometre.

For a better understanding of our experimental findings, we calculated the plasmon resonances of the individual nano-triangles and computed a convolution of the response when scanning Fischer patterns through a laser focus (Figure 7). We theoretically modelled the imaging process by assuming a three dimensional triangle structure similar to the true geometry of one basic unit of the Fischer patterns. The size of the triangle can be tuned according to the Fischer patterns used in the experiments. The triangle is positioned at different positions within the focus of either an azimuthally or a radially polarised laser beam to simulate the imaging collection process in a scanning optical microscope. The field strength distributions in the x,y-plane and the z-plane of either an azimuthally or radially polarised beam focused by a parabolic mirror are calculated according to literature [24]. The wavelength-dependent extinction spectrum of one nano-triangle is calculated using the ‘surface integral equation’ method [30]. The response of the triangle to the focus field is then multiplied according to the number of nano-triangles within the scanning range, yielding a convolution image from many Fischer patterns. The optical signal counted by this model is based on the proportionality of the luminescence signal to the incident electric field intensity. Due to the high numerical aperture (NA = 0.9998) almost all the luminescence signal is collected. Scanning the structure through the focal plane consequently corresponds to a convolution of the structure with the field intensity (as depicted in Figure 1b,c) in the focal plane.

When comparing the experimentally obtained luminescence patterns of gold nano-triangles on silicon (Figure 3 and Figure 4) to the theoretical convolution images, we found several matches: the luminescence patterns of Fischer patterns with *h* = 200 nm are reproduced by the calculations. However, comparing the experimental images of the nano-triangles on glass (Figure 5 and Figure 6) to the convolution patterns, we found that the discrepancy is larger. The mismatches between our experimental results and the model originate from the following reasons: 1) No substrate is considered in the model, therefore, the influence from the dielectric materials such as the glass and silicon is inevitably missing. 2) The foci at the air/silicon and air/glass interfaces are different due to the different material properties. 3) This simple convolution approach accurately describes the scattering at single nanostructures but completely neglects the coupling between the elements of the Fischer pattern, both in the form of near-field coupling and through diffraction. However, as seen in Figure 2, in some of the cases, the triangles nearly touch. Therefore, more parameters will be considered in future simulations. Diffraction due to the Fischer patterns' periodicity may in fact cause a discrepancy between the simulated and measured images. Diffraction is important particularly when many scatterers are involved, e.g., under plane wave illumination. In our case however, only single or few particles are illuminated with a tightly focused laser beam [24] and hence, diffraction is expected to give minor errors when comparing the convolution patterns to the experimental results. 4) Furthermore, Marti et al. have recently revealed small clusters formed at the edges of triangular particles from high resolution SEM imaging [31]. In the gaps between small spherical particles and large triangular particles, highly concentrated near fields can be confined. Such a phenomenon can also contribute to the differences between our experimental images and the theoretical convolution.

### 3. Surface-enhanced Raman spectra

Spectroscopic methods have been applied to study light-matter interaction at nanometre scale or single molecule level [32]. Different from fluorescence spectroscopy, Raman spectroscopy provides a unique fingerprint of the molecules and therefore does not require labelling of the samples. However, conventional Raman spectroscopy is an ensemble approach where locally and temporally restricted effects will get smeared out and weak signals will easily be covered by stronger ones. Biological systems – such as DNA-drug interactions – give such subtle signals. However, for their study more sensitive and selective methods, which ideally measure on a single molecular level, are required. Conventional Raman spectroscopy fails at such low concentrations because of the feebleness of the inelastically scattered light. Surface-enhanced Raman scattering (SERS) is a possible solution to this problem.

**Figure 8:**
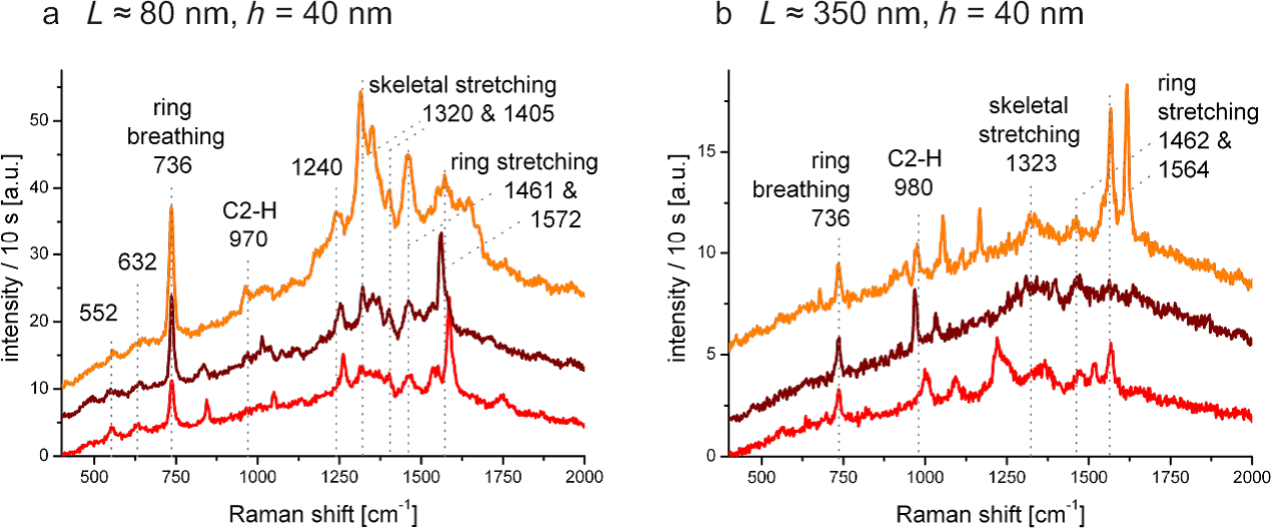
Surface-enhanced Raman spectra of an adenine (sub-)monolayer on gold Fisher patterns on glass. The nano-triangles which were used had an edge length of (a) *L* ≈ 80 nm and (b) *L* ≈ 350 nm. In both cases, their height was *h* ≈ 40 nm. Three spectra, offset for clarity, are shown in both cases to demonstrate the reproducibility of the measurements at different spots. All spectra were recorded in radial donut mode (λ = 632.8 nm, focal power 250 µW) and with an acquisition time of 10 s.

Ultimately, we assessed the gold Fischer patterns' applicability as SERS enhancing substrates [17] by letting adsorb a (sub-)monolayer of the DNA base adenine. As expected by the confocal studies where the triangles on glass had shown a stronger luminescence and a higher contrast, these now proved to be better suited substrates for SERS. Especially the thin triangles (*h* ≈ 40 nm) on glass yielded reproducible SERS spectra in radial mode (Figure 8). With both of these Fischer patterns (with *L* ≈ 80 and *L* ≈ 350 nm), the typical ring breathing mode of adenine was clearly observed and was highly reproducible at 736 cm^−1^. Further bands could be assigned according to previous SERS measurements in literature [33–37].

Between individual spectra, slight fluctuations were observed, indicating that only a very low amount of molecules was probed. We believe these differences to stem from varying adsorption positions of the molecules, e.g., on the flat top part of a triangle or at its edge. The most efficient Raman enhancement occurs when the transition moment of a molecular excitation is parallel to the electric near-field component [38], which results in the favouring of different Raman bands in these molecules. Using an azimuthally polarised beam, SERS measurements of adenine adsorbed on Fischer patterns (*L* ≈ 350 nm, glass substrate) showed similar Raman bands as those observed with a radially polarised beam; however, the intensities are much weaker (Supporting Information File 1). Due to the extremely low surface coverage of DNA molecules, no Raman peak is observable from a smooth gold thin film on which no Fischer patterns are present (Supporting Information File 1). Our results indicate that Fischer patterns can be used as effective SERS substrates for optical sensors based on the plasmonic near-field enhancing effect.

## Conclusion

The plasmonic excitations of Fischer patterns can be imaged in a simple confocal setup using a parabolic mirror and employing higher order laser modes. We investigated gold Fischer patterns on two different substrates. The obtained luminescence patterns varied strongly regarding on the aspect ratio of the nano-triangles, the excitation by either radial or azimuthal donut mode and the underlying substrate. In general, glass as a substrate yielded images with higher contrast compared to silicon which in several cases returned no detectable contrast at all. Additionally, we noticed that larger gold areas due to defects yielded regions of weaker luminescence. We consider this a proof that the strong luminescence stems from efficient excitation of the nano-triangles. A comparison with a simple theoretical convolution of the field strength in the focus and the layout of the triangles is made. To minimise the discrepancies, more extensive simulations are undergoing.

When depositing adenine molecules on the gold triangles on glass, we obtained strongly enhanced Raman spectra which can clearly be assigned to the different vibrational bands of adenine. We thereby proved the usability of gold Fischer patterns on glass as suitable SERS substrates. Since Fischer patterns can easily, cheaply and reproducibly be fabricated on large areas, we advocate that they be used as effective substrates for biosensors based on plasmonic near-field enhanced spectroscopy.

## Supporting Information

File 1 SERS and Raman spectra of adenine molecules.
