# Supplementary material for: Assessing the plasmonics of gold nano-triangles with higher order laser modes
Source: Beilstein J Nanotechnol. 2012 Oct 4;3:674–83. doi: 10.3762/bjnano.3.77 (PMC3512117; doi:10.3762/bjnano.3.77)
Supplement: File 1 — SERS and Raman spectra of adenine molecules. [file Beilstein_J_Nanotechnol-03-674-s001.pdf]

Supporting Information

for

Assessing the plasmonics of gold nano-triangles with higher order laser modes

Laura E. Hennemann[1], Andreas Kolloch[2], Andreas Kern[1], Josip Mihaljevic[1], Johannes Boneberg[2], Paul Leiderer[2], Alfred J. Meixner[1] and Dai Zhang*[1]

Address: [1]Institute of Physical and Theoretical Chemistry, University of Tübingen, Auf der Morgenstelle 15, 72076 Tübingen, Germany and [2]Department of Physics, University of Konstanz, Universitätsstrasse 10, 78457 Konstanz, Germany

Email: Dai Zhang - dai.zhang@uni-tuebingen.de

* Corresponding author

SERS and Raman spectra of adenine molecules

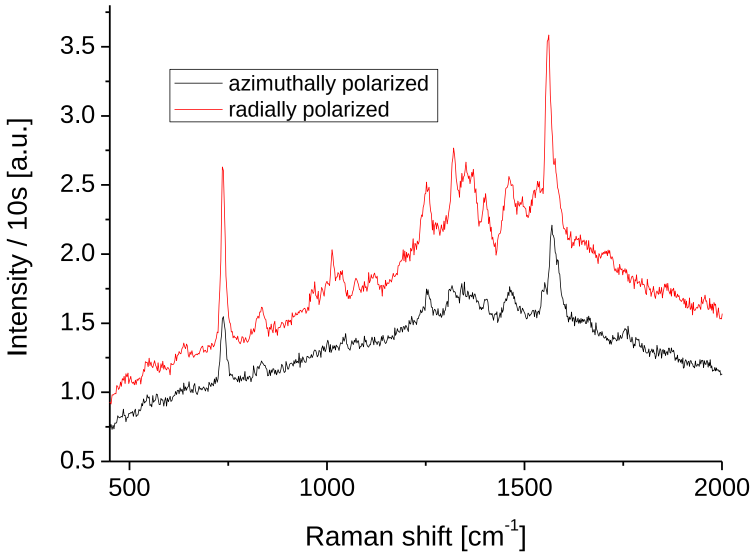

Figure S1: SERS spectra of adenine molecules adsorbed on a gold Fischer pattern (*L* ≈ 350 nm, glass substrate). The black spectrum was collected through excitation with an azimuthally polarised laser beam. The red spectrum was collected through excitation with a radially polarised laser beam.

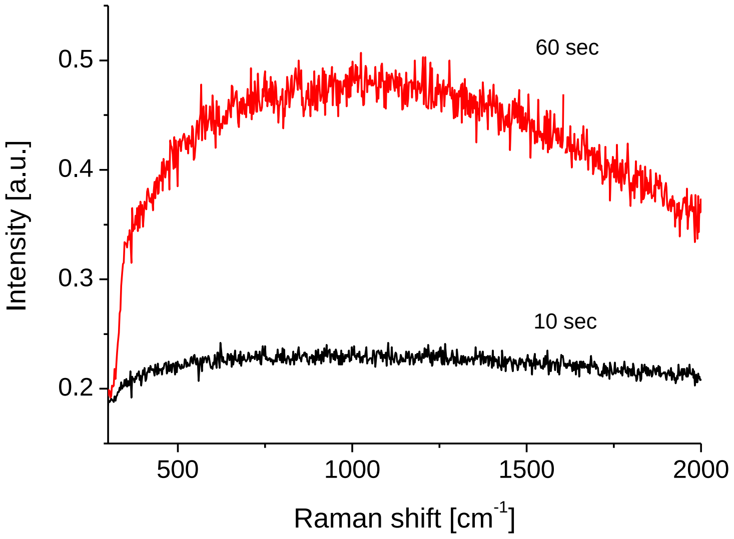

Figure S2: Raman spectra of adenine molecules adsorbed on a gold thin film using two different integration times. No obvious Raman peaks are observable due to the extremely low coverage with molecules. Both spectra were collected in radial donut mode.
